# Credibility and advocacy in conservation science

**DOI:** 10.1111/cobi.12558

**Published:** 2015-08-28

**Authors:** Cristi C. Horton, Tarla Rai Peterson, Paulami Banerjee, Markus J. Peterson

**Affiliations:** ^1^ Department of Communication StudiesTarleton State UniversityStephenvilleTX 76402U.S.A.; ^2^ Department of CommunicationUniversity of Texas at El PasoEl PasoTX 79968U.S.A.; ^3^ Department of Urban and Rural DevelopmentSwedish University of Agricultural SciencesUppsala750 07Sweden; ^4^ Department of Biological SciencesUniversity of Texas at El PasoEl Paso TX 79968U.S.A.; ^5^ Program in Environmental Science and EngineeringUniversity of Texas at El PasoEl PasoTX79968U.S.A.

**Keywords:** advocacy, communication, content analysis, credibility, environmental policy, grounded theory, oblique component cluster analysis, rhetoric, abogacía, análisis clúster de componentes oblicuos, análisis de contenido, comunicación, credibilidad, política ambiental, retórica, teoría fundamentada

## Abstract

*Conservation policy sits at the nexus of natural science and politics. On the one hand, conservation scientists strive to maintain scientific credibility by emphasizing that their research findings are the result of disinterested observations of reality. On the other hand, conservation scientists are committed to conservation even if they do not advocate a particular policy. The professional conservation literature offers guidance on negotiating the relationship between scientific objectivity and political advocacy without damaging conservation science's credibility. The value of this guidance, however, may be restricted by limited recognition of credibility's multidimensionality and emergent nature: it emerges through perceptions of expertise, goodwill, and trustworthiness. We used content analysis of the literature to determine how credibility is framed in conservation science as it relates to apparent contradictions between science and advocacy. Credibility typically was framed as a static entity lacking dimensionality. Authors identified expertise or trustworthiness as important, but rarely mentioned goodwill. They usually did not identify expertise, goodwill, or trustworthiness as dimensions of credibility or recognize interactions among these 3 dimensions of credibility. This oversimplification may limit the ability of conservation scientists to contribute to biodiversity conservation. Accounting for the emergent quality and multidimensionality of credibility should enable conservation scientists to advance biodiversity conservation more effectively*.

## Abstract (pt)

Credibilidad y Abogacía en la Ciencia de la Conservación

**Resumen**

*La política de la conservación se encuentra en el vínculo entre la ciencia natural y la política. Por un lado, los científicos de la conservación pugnan por mantener la credibilidad científica enfatizando que sus resultados de investigaciones son el resultado de observaciones desinteresadas de la realidad. Por el otro lado, los científicos de la conservación están comprometidos con la conservación aun cuando no tienen que promover una política determinada. La literatura profesional de conservación ofrece guías para negociar la relación entre la objetividad científica y la defensa política sin dañar la credibilidad de la ciencia de la conservación. Sin embargo, el valor de esta guía puede ser restringida por el reconocimiento limitado de la multidimensionalidad y naturaleza emergente de la credibilidad: emerge a través de percepciones de conocimiento, buena voluntad y confiabilidad. Utilizamos el análisis de contenidos de la literatura para determinar como se enmarca la credibilidad en la ciencia de la conservación ya que se relaciona con contradicciones aparentes entre la ciencia y la abogacía. La credibilidad típicamente se enmarcó como una entidad estática carente de dimensión. Los autores identificaron el conocimiento o la confiabilidad como importante, pero raramente mencionaron la buena voluntad. Generalmente, no identificaron el conocimiento, la buena voluntad o la confiabilidad como dimensiones de la credibilidad ni reconocieron las interacciones entre estas 3 dimensiones de la credibilidad. Esta simplificación excesiva puede limitar la habilidad de los científicos de la conservación para contribuir a la conservación de la biodiversidad. El tomar en cuenta la cualidad emergente y la multidimensionalidad de la credibilidad deberá permitir que los científicos de la conservación avancen más efectivamente en la conservación de la biodiversidad*.

## Introduction

Conservation policy sits at the nexus of natural science and politics. Conservation scientists practice a crisis discipline driven by overt values that require them to juggle the roles of providing objective information about the natural world and advocating policies and approaches likely to promote biodiversity conservation (Soulé 1985, 1986). Most applied ecologists realize policy decisions are often made before scientific evidence is complete (Kinchy & Kleinman 2003; Morrison et al. 2008; Peterson 2009). Risks to biodiversity exacerbate this situation, requiring conservation scientists to act before they are confident in the sufficiency of their data because time is of the essence (Soulé 1985, 1986; Brussard & Tull 2007). Therefore, a conundrum grows out of the relationship among scientific expertise, advocacy, and credibility (Kinchy & Kleinman 2003). On the one hand, both the lay public (Jasanoff et al. 1995) and many natural scientists (Morrison et al. 2008; Peterson 2009) typically assume that credible knowledge produced by science emerges from disinterested observations of objective reality (Popper 1959, 1962; Platt 1964). On the other hand, conservation scientists are committed to some form of biodiversity conservation even if they do not advocate a particular policy (Naess 1986; Peterson 2009; Meyer et al. 2010). This means they simultaneously play the apparently paradoxical roles of scientist and advocate—a situation bound to produce dissonance, given current social constructs. Recognition of this perceived conundrum has prompted sustained discussion of scientific credibility in the conservation literature. We analyzed that discussion and suggest ways to fortify its contributions.

Systematic analysis of credibility dates at least to the fourth century BCE with Aristotle's *Rhetoric* (4th century BCE/1991), which argues that the most effective persuasion combines situationally appropriate logical, emotional, and ethical appeals. In contemporary parlance, ethical appeals refer to the construction of credibility (Kennedy 1999). Aristotle described credibility as emerging from the dimensions of expertise, goodwill, and trustworthiness (Aristotle 4th century BCE/1991; Kennedy 1999). Expertise refers to specialized knowledge a person possesses on a subject and is often embodied in credentials or special skills obtained from training or education. Goodwill describes caring for others’ well‐being and is demonstrated by empathy developed by direct interaction with others. Trustworthiness refers to the person's honesty. Trustworthy persons demonstrate integrity, are unbiased, and absolutely honest. Although credibility is associated with perceptions of a communicator's character, it does not exist within an individual or an organization; rather, it is jointly constructed (Moon & Blackman 2014). Thus, credibility, as well as any of its dimensions, may be interpreted differently, depending on who is participating in the communicative event.

Credibility is more of a relational property than a static entity (Rhee & Fiss 2014), attaining relative stability only when it functions as an “attitude toward a source of communication held at a given time by a receiver” (McCroskey 1997:87). The most productive credibility emerges from situationally appropriate integration of expertise, goodwill, and trustworthiness (Aristotle 4th century BCE/1991; Burke 1966; Kennedy 1999). For example, in some cases a scientist's impeccable credentials (i.e., PhD, publication record, etc.) may be less important to her or his credibility than demonstrated willingness to join with community members in their efforts to ensure that the effects of a drought do not extinguish a small population of endangered Attwater's Prairie‐Chickens (*Tympanuchus cupido attwateri*) (Peterson & Silvy 1994, 1996; Silvy et al. 2004). In other situations, such as determining whether to list a species as endangered, impeccable credentials may be the most important factor in credibility. As a perceptual construct, credibility is based on social relations and is co‐constructed within each situation, which creates a set of expectations. For example, when ranchers are told they may not clear woody vegetation because their pasture land has been designated critical habitat for an endangered species, much of their anger stems from the violation of expectations regarding private property rights (Peterson & Horton 1995). For these reasons, deciding which dimension or dimensions of credibility will be most useful for avoiding the violation of expectations requires careful assessment of each situation (Cronkhite & Liska 1976). What is generalizable across situations is that participant expectations vary according to cultural, economic, and political aspects of a situation and the credibility that participants attribute to an individual or organization emerges largely from whether their expectations are fulfilled (Burke 1966; Cronkhite & Liska 1976).

Contemporary social science research on credibility examines both its multidimensionality and its emergent nature, often with similar objectives as found in the conservation literature. Rhee and Fiss (2014), for example, examined how interactions between language and different dimensions of a communicator's credibility encouraged and discouraged organizations to undertake controversial actions. They found that promotion of risky activities by persons who appeared to lack goodwill discouraged organizations from undertaking such activities. Malshe (2010) studied interactions between the three dimensions of credibility (i.e., expertise, goodwill, trustworthiness) to help managers handle relations between marketers and salespeople. He demonstrated that understanding the sometimes incompatible interactivity between these three components expanded management's repertoire of tools for harmonizing internal business interactions.

A situationally nuanced understanding of credibility is especially important to conservation scientists because, as an act of dynamic progression, credibility is largely contingent on situational aspects that contribute to or reduce the satisfaction of participant expectations. It is not static and is subject to patterns of language or terminologies that provide people with socially accepted ways to represent and constitute reality (Burke 1966; Peterson et al. 2013:94). Conservation professionals have an opportunity to enhance their credibility by consistently using terminologies that contribute to public expectations, and later, satisfy those expectations (Newell & Goldsmith 2001; Kao 2013).

Conservation science literature recognizes the importance of credibility, yet struggles with tensions between science and advocacy. Recognizing that credibility matters is not the same thing as understanding how it emerges and operates. We analyzed the professional conservation science literature to identify the primary points of guidance offered to conservation professionals regarding how to manage their responsibilities as scientists and advocates. For this report, we first determined which dimensions of credibility the literature emphasized when describing conservation scientists’ credibility. Next, we identified relative concern about risks to biodiversity, professional credibility, and sustainability. Third, we identified the preferred roles this literature suggested conservation professionals should play. We then explored how the literature defined conservation science. Finally, we determined how the professional literature described a credible environmental policy process. After coding for each of these variables, we explored the relationships among them and considered the importance of the multidimensionality of credibility and its dependence on sociopolitical context.

## Methods

We used a grounded theory approach (Corbin & Strauss 2008) to guide our content analysis of the professional conservation science literature. This refers to a process of allowing a theoretical framework to emerge from the data (Peterson et al. 2010). We began with articles from a special issue of *Conservation Biology* that discussed the relationship between policy advocacy and conservation science (i.e., Brussard & Tull 2007; Lackey 2007; Meffe 2007; Murphy & Noon 2007; Noss 2007; Scott et al. 2007). The key terms used to address this enigmatic relationship were advocacy, opinion, and scientific independence. Using these terms, we searched the ISI Web of Knowledge for refereed journal articles in the field of conservation biology from 1990 through 2010. We found 30 articles of which 11 were relevant to the scientist–advocate dilemma. We then read each article closely (Leff 1980) to identify additional key terms to guide an expanded search. Based on the close readings, we selected 7 additional terms (*conservation*, *credibility*, *expert opinion*, *neutrality*, *science impartiality*, *science integrity*, *subjectivity*).

Next, we searched the ISI Web of Knowledge, Google Scholar, Wiley Online Library, and Discovery databases for the terms we identified in titles, keywords, and abstracts of refereed journal articles and book chapters published 1976–2012. In publications that lacked keywords or abstracts, we searched the entire document. We carefully read each publication and removed those not directly relevant to perceived trade‐offs between science and advocacy. This process yielded an additional 119 publications for a total of 136.

We identified common themes across the conservation science literature (Peterson et al. 1994; Peterson et al. 2010) to create categories that captured the concepts used to explore the fraught relationship between science and advocacy. We used constant comparison (Corbin & Strauss 2008) between preexisting and emerging categories. We raised questions regarding formulation of categories and documented and analyzed ideas about categories as they were refined. Saturation (no new categories emerged) was reached after creating 5 categories and 12 subcategories (Table 1). In addition to credibility, the categories that emerged were risk (What are conservation biologists most worried about?), role (How should conservation scientists engage the issues?), conservation science (What does conservation science include?), and environmental policy (What should policy be based on?).

**Table 1:** Categories and sub‐categories used for content analysis of conservation science publications (*n* = 136; 1976–2012) discussing tensions between science and advocacy

Category	Subcategory	Definition
Credibility	expertise	conservation scientists’ specialized knowledge
	goodwill	conservation biologists’ care for natural resources and society
	trustworthiness	conservation biologists’ integrity
Conservation science	intersubjective	conservation of biological diversity is in part a social process that includes values and argumentation
	objective	conservation of biological diversity is evidence‐based science
Environmental policy process	natural science	environmental policy is based only on natural science
	social and natural science	environmental policy is based on natural science and important social aspects (economics, law, politics)
Risk	biodiversity	all aspects of variety in the living world
	scientific credibility	conservation biologists (believability and standing)
	sustainability	ecosystems and their functions
Role	advise, report, or both	educate in the policy realm, provide data results, or both
	advocate	support a preferred policy or practice

Our methods and results were iteratively linked (Corbin & Strauss 2008), such that each category and subcategory that emerged during content analysis contributed to refinement and clarification of already existing categories (see Content Analysis, in Results, for linkage details). When authors explicitly discussed credibility, we examined the sentence to determine relative emphasis on expertise, goodwill, and trustworthiness (Table 1). We developed a codebook which defined categories and subcategories and then used it to train coders and assess intercoder reliability (Krippendorff 2013). Coders used NVivo 10.0 qualitative software (QSR International, Doncaster, Victoria, Australia) to code publication abstracts. For publications without abstracts, we coded the publications’ introduction or conclusion (hereafter summaries) depending on which one best summarized the content. Sentences were the unit of analysis. The same sentence was coded in multiple categories if it fit more than one. Two people independently coded all abstracts and summaries. We calculated intercoder reliability across all summaries and categories with weighted Cohen's kappa (Cohen 1968; κ = 0.8756).

Our final analytic objective was to explore relationships among the 12 subcategories (i.e., variables) delineated through content analysis (Table 1). Because procedures such as principle component and factor analysis produce principle components and factors, respectively, that include information from all variables, we used oblique component cluster analysis to group variables in SAS 9.3 (VARCLUS procedure; SAS Institute 2012). This procedure iteratively reassigns variables to clusters such that variance explained by cluster components, summed over all clusters, is maximized. We stopped iterative clustering once the largest second eigenvalue dropped below 0.95.

## Results

### Content Analysis

**Figure 1:**
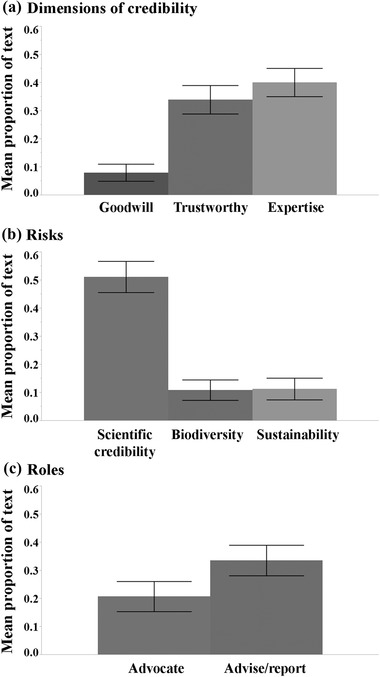
Mean (95% CI) proportion of evaluated conservation science text coded during content analysis as relevant to (a) the dimensions of credibility (goodwill, trustworthiness, and expertise), (b) risk to individuals’ scientific credibility, biodiversity, and sustainability of ecosystems and their functions, and (c) the role of conservation scientists as advocates or advisers and reporters or both.

**Figure 2:**
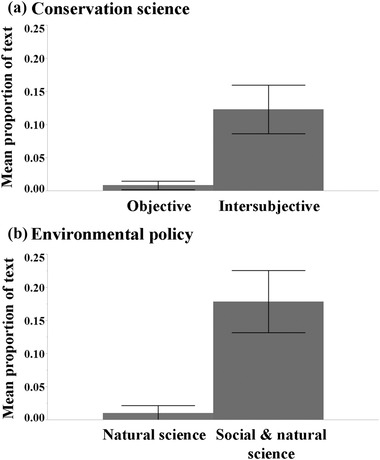
Mean (95% CI) proportion of evaluated conservation science text coded during content analysis maintaining that (a) conservation science is an objective versus an intersubjective enterprise and (b) the environmental policy process should be grounded in natural science versus both social and natural science.

All our findings related in some way to credibility. When authors discussed credibility, 40.2% and 34.0% of the text evaluated, on average (*n* = 136 publications), addressed expertise and trustworthiness, respectively, rather than goodwill (8.1%) (Fig. 1a). The majority of statements describing risks focused on concern about loss of scientific credibility, rather than risks to biodiversity or sustainability ($\bar{x}$= 51.2% versus 11.0% and 11.4% of text evaluated, respectively) (Fig. 1b). As authors considered the roles conservation scientists should play in the conservation policy arena, they emphasized educating the public and policy makers or providing data to policy makers rather than advocating for particular conservation actions ($\bar{x}$ = 33.6% versus 20.8% of text evaluated, respectively) (Fig. 1c). When authors discussed conservation science, they described it as including social processes rather than being limited to evidence‐based natural science ($\bar{x}$= 12.3% versus 0.9% of text evaluated, respectively) (Fig. 2a). Finally, statements about environmental policy centered on the claim that the policy process involves natural science and important social components—including economics, politics, and law—as contrasted with the notion than environmental policy should be based strictly on natural science ($\bar{x}$= 18.0% versus 1.1% of text evaluated, respectively) (Fig. 2b).

References to the value of conservation scientists’ specialized knowledge as a means of enhancing credibility demonstrate attention to credibility as it is associated with expertise (hereafter credibility‐expertise) (Table 1). For example, the statement “Nobody is suggesting that conservation scientists should always and consistently shy away from policy and never lend their expertise to public issues” (Meffe 2007:11) illustrates this concern. Recommendations that conservation scientists should care for natural resources indicates concern with credibility as it relates to goodwill (credibility‐goodwill). Examples include phrases such as “Wildlife managers are stewards of a public resource” (Decker et al. 1991:526) and “We have little choice if we truly want to conserve that diversity for its inherent good” (Meffe & Viederman 1995:331). Authors sometimes refer to conservation scientists’ integrity or credibility as it relates to trustworthiness (credibility‐trustworthiness). For example, the statement “Budgetary dependence of state wildlife agencies on…license fees automatically raises concerns about their ability to act fairly” (Rutberg 2001:33) illustrates a focus on credibility‐trustworthiness.

Risks of various sorts are important aspects of credibility for conservation scientists (Table 1). The phrase “the accelerated loss of biodiversity” (Vohland et al. 2011:1188) indicates an emphasis on risks to biodiversity (risk‐biodiversity), whereas the phrase “because of the increasing consequences of the alteration of biotic systems” (Mooney 2003:49) exemplifies concern with sustainability of ecosystems and their functions (risk‐sustainability). Statements referring to conservation scientists’ loss of standing or believability illustrate awareness of risks to scientific credibility (risk‐scientific credibility). For example, the sentence “[S]cientists who lack impartiality often create the perception of bias, and they can suffer a concomitant loss of credibility” (Ruggiero 2010:1179) demonstrates concern with risk‐scientific credibility. This sentence also illustrates the possibility of assigning multiple codes to a single sentence because its reference to “lack [of] impartiality” indicates concern with credibility‐trustworthiness.

The literature suggested that conservation professionals could enhance their credibility by playing appropriate roles. When authors indicated the appropriate role for conservation scientists was to educate and provide data, they tended to direct their colleagues toward an advisory role, which primarily consists of reporting on the results of their research (Table 1). For example, the phrases “policymakers, managers, and the lay public need scientific counsel all the more” (Allen et al. 2001:484) and “they [scientists] should inform the public about issues while avoiding direct involvement in policy development” (Ruggiero 2010:1179) suggest that conservation professionals should limit their policy involvement to the role of advising or reporting. Some authors, however, recommended that conservation scientists support specific policies, taking the role of advocate. For example, the sentence “[T]he question is not whether we should advocate but how” (Chan 2008:3) recommends that conservation professionals have a responsibility to function as advocates.

Many authors described conservation science as a social process that includes values and argumentation, resulting in a code of conservation science‐intersubjective (Table 1). The sentence “Conservation biologists should reflect on the constitutive values (especially contextual, but also methodological and bias) underlying their research programs” (Barry & Oelschlaeger 1996:905) typifies statements that described conservation science as intersubjective. Other authors state that conservation science should be based strictly on empirical evidence because conservation science should be objective. For example, consider the text “[I]t is imperative to understand the distinction between science and professional judgment. The former is the acquisition of knowledge by applying the principles of the scientific method” (Sallenave & Cowley 2006:203). It illustrates such claims and was coded as conservation science‐objective.

Authors presented similarly divergent arguments regarding what led to excellent environmental policy. Some claimed it should be based only on natural science (Table 1). For example, the statement that “Environmental policies and actions can be improved…by calling attention to relevant scientific information and ensuring that policies and their implementation are consistent with the best available science” (Meyer et al. 2010:299) represents environmental policy process focused on natural science (policy‐natural science). Alternatively, sentences that explain appropriate environmental policy as based on the integration of natural (e.g., ecology) and social sciences (e.g., economics, law, politics) demonstrate the focus on social and natural science in environmental policy (policy‐social and natural science). Text such as “identification of visionary science questions…and identification of questions about human values and their role in political processes could all help advance real‐world conservation science” (Rudd 2011:860) illustrates a preference for policy‐social and natural science.

### Interactions among Categories

When authors discussed credibility, risks, and the roles conservation scientists should play, there were recognizable interactions among the themes they emphasized. Authors who emphasized credibility as trustworthiness were quite concerned about risks to their scientific credibility and claimed conservation science should be an objective enterprise (Table 2, cluster 1). Statements such as “[B]ias…associated with lobbying efforts all tend to dissuade scientists from participation as advocates…[but] the presentation of relevant data and insistence that it be interpreted accurately and acted upon is an effective method of achieving biologically sound policies” (Salzman 1989:170) illustrate the claim that conservation scientists’ integrity (trustworthiness) is essential to their believability when practicing evidence‐based conservation science. Authors who used scientific expertise to define credibility also discussed risks to ecological sustainability and claimed the primary roles conservation scientists should play were assessing data, reporting results, and advising the public and environmental policy makers (Table 2, cluster 2). For example, the text “[D]evelopment of new laws and policies must account for uncertainties…and complexities of ecological systems…” and “Scientists need to recognize that…the results of fundamental research can contribute greatly to the use of sound ecological principles in legislation and policy” (Brosnan 1995:333) indicates that expertise is important to legitimizing the preferred role of advisor or reporter. Finally, when authors defined credibility as goodwill—or acting in the interest of the resource and society—they also were concerned about risks to biodiversity (Table 2, cluster 3). “The vast majority of those who call themselves conservation biologists were attracted to their field out of a love for nature.… Scientific knowledge and understanding will help us to be more successful in our common goal of preserving global biodiversity” (Tracy & Brussard 1996:918) illustrates that goodwill is intrinsic to any effort to curtail the continued loss of species, communities, and ecosystems.

**Table 2:** Iterative oblique component cluster analysis results for content analysis variables for publications^a^ discussing the tensions between science and advocacy (proportion of total variance explained by variable clustering = 0.572)

Cluster	Category^b^	Subcategory^b^	*R* ^2^ own cluster	*R* ^2^ next closest
1	credibility	trustworthiness	0.657	0.014
	risk	scientific credibility	0.625	0.058
	conservation science	objective	0.364	0.041
2	credibility	expertise	0.645	0.050
	role	advise, report, or both	0.638	0.109
	risk	sustainability	0.189	0.023
3	risk	biodiversity	0.629	0.015
	credibility	goodwill	0.629	0.034
4	environmental policy	natural science	0.612	0.002
	role	advocate	0.612	0.036
5	conservation science	intersubjective	0.632	0.044
	environmental policy	social and natural science	0.632	0.081

a *Number of publication,136; years of publication, 1976–2012*.

b *See Table* 1 *for definitions*.

Interactions among themes also emerged when authors discussed environmental policy. Authors who argued that environmental policy should be grounded almost exclusively on evidence‐based natural science claimed that conservation scientists should indeed play the advocate role in the policy process (Table 2, cluster 4). For example, the statement “involvement in developing…conservation policy is an important activity that more wildlife professionals should become comfortable with as objective advocates for science‐based policy” (Thompson 1995:318) suggests that conservation professionals should advocate for specific conservation policies so long as their advocacy is based on objective natural science. Authors who maintained that environmental policy must be grounded on both social and natural science argued that conservation science is an intersubjective rather than a strictly objective discipline (Table 2, cluster 5). Statements such as “how they [science and policy] fit together is best understood by viewing land management as a process [that clarifies]…why it is proper for conservation biologists to base their work on normative goals” (Freyfogle & Newton 2002:863) illustrate that for these individuals, conservation science encompasses evidenced‐based natural science, social science, and social interactions.

## Discussion

Credibility is a slippery shibboleth (Macnab 1985) for conservation scientists. As Alagona (2008:1365) put it, “everybody seems to think credibility is a good idea…. But exactly what credibility is remains the subject of considerable confusion.” Various pairings of the term, such as *scientific credibility* (Costanza 2001:459; Wilhere 2012:40), *professional credibility* (Gill 2001:22), and *agency credibility* (Rutberg 2001:33), contribute to the confusion. Occasionally, the literature defines credibility as believability or as inspiring trust (Blockstein 2002; Nelson & Vucetich 2009; Ruggiero 2010; Yamamoto 2012). But, as noted in the Introduction, such definitions are incomplete characterizations of credibility. Although some credibility research uses a 2‐dimensional model of credibility that collapses goodwill and trustworthiness into a single item for measurement purposes (Newell & Goldsmith 2001), the distinction is important to maintain when considering credibility in conservation science. Although, in some cases, a scientist's perceived honesty may be the primary contributor to her or his credibility, in other situations it may be far more important to demonstrate that the scientist cares about human welfare as well as biodiversity.

### Confusion about Credibility

We found that the conservation science literature does not present a multidimensional picture of credibility. The publications we analyzed demonstrated a lack of awareness that credibility develops along the dimensions of expertise, goodwill, and trustworthiness delineated by Aristotle (4th century BCE/1991; Kennedy 1999) and further studied by contemporary social scientists (e.g., Malshe 2010; Kao 2013; Sah et al. 2013; Rhee & Fiss 2014). These dimensions are either omitted or listed as entities that exist separately from credibility (e.g., Blockstein 2002; Goodwin 2012). We submit that conservation scientists could more effectively enhance their credibility by emphasizing appropriate combinations of these dimensions in response to situational demands. For example, when discussing potential changes in the legal status of the federally endangered Golden‐cheeked Warbler (*Setophaga chrysoparia*) with decision makers, they would most likely need to emphasize their expertise. Conservation scientists also should recognize and respond to opportunities to use a powerful combination of two or more credibility dimensions (Table 2, clusters 1–3). For instance, if they are interacting with bird watchers concerned that Golden‐cheeked Warbler habitat is being destroyed on public property, they would most likely need to highlight both their expertise and trustworthiness, with goodwill being assumed. Conversely, if conservation professionals are interacting with ranchers concerned that their agriculture livelihood is threatened because their property has been designated as critical habitat for the endangered species, conservation scientists would most likely need to demonstrate goodwill, allowing trustworthiness and expertise to take a backseat.

The conservation literature we analyzed typically framed credibility as a static entity, rather than a social construct that depends on precarious, but quite real, social relationships (Aristotle 4th century BCE/1991; Burke 1966; Kennedy 1999). Conservation scientists can improve their ability to discover the most important dimensions of credibility in each situation if they remember that humans understand the world from within their own sense of self. Preexisting values and beliefs give meaning to new experiences, which then modify those values and beliefs. This iterative process leads to expectations that people use to judge any message, policy, or action and that influence credibility in any sociopolitical context. For example, if birders have been involved in successful citizen–science projects, they are likely to assume goodwill from conservation professionals, freeing scientists to focus on demonstrating their expertise and trustworthiness. Because it is a perceptual construct, conservation scientists have only partial control over credibility. They can enhance their credibility by engaging with stakeholders to determine what it means to be credible, working toward achieving that credibility, and then behaving as credibly as possible given the demands of each situation.

### Recognizing Credibility's Multidimensionality

The overly simplistic and unidimensional framing of credibility in the conservation literature limits the value of advice about how risk and roles contribute to, and potentially damage, credibility. For example, although conservation scientists are alarmed about risks to biodiversity and sustainability, these concerns are overshadowed by risks to their professional credibility (Figure 1b). A more nuanced understanding of credibility would provide a means for assessing which dimensions of credibility are most important in each situation. For example, the preferred role of advisor or reporter (Fig. 1c) clustered with expertise as the means for addressing risks to sustainability (Table 2, cluster 2). In some situations, however, the trustworthiness or goodwill dimensions may be more credible ways to address sustainability risks than expertise. Conservation professionals sometimes must play an advocacy role, and risks to biodiversity and sustainability may trump risks to professional credibility. Awareness of multiple possibilities for enhancing credibility by strategic role taking could contribute directly to successfully negotiating whichever risk requires the most immediate attention in a given situation (Table 2).

An oversimplified framing of credibility also limits the value of advice regarding how to best use conservation science to inform policy. The conservation literature we evaluated linked the trustworthiness dimension of credibility with risks to professional credibility and the claim that conservation science should be objective (Table 2, cluster 1). This suggests that professional credibility depends on accepting the premise that conservation science should be an objective enterprise uncoupled from social values. However, any momentary condition of credibility results from complex sociopolitical processes that operate recurrently (although not necessarily consistently) and that are socially constructed (Aristotle 4th century BCE/1991; Burke 1966; McCroskey 1997). Despite the relationships identified in cluster 1, the professional literature characterizes conservation science as intersubjective (Fig. 2a) and as the basis for environmental policy (Fig. 2b). These close connections indicate an understanding that both conservation science and environmental policy include sociopolitical aspects (Moon & Blackman 2014) that extend well beyond the material world into peoples’ relationships with Earth (Table 2, cluster 5). A more complete understanding of how credibility develops should enable conservation scientists to build on this awareness by explicitly emphasizing the appropriate dimensions of credibility in each situation.

Understanding the multidimensionality of credibility and recognizing it as a relational property, rather than as a static entity, should help conservation scientists make appropriate choices for legitimizing the various roles they play. Returning to the example of conservation scientists communicating with ranchers who have interests in management of Golden‐cheeked Warbler habitat, the role of advocate would likely be completely inappropriate, whereas the role of advisor or reporter may be acceptable. Downplaying their expertise is one way conservation scientists can signal respect for ranchers’ local experiential knowledge, which is especially important if the ranchers are feeling nervous about potential inroads into their property rights (Peterson & Horton 1995). Conversely, if conservation scientists think their findings indicate that the species has made significant strides toward recovery, they may decide to step into an advocacy role, suggesting that the U.S. Fish and Wildlife Service downlist the species to threatened. In this situation, conservation scientists might want to emphasize their expertise and complement this with indications that they are unbiased, or trustworthy. For this stakeholder group and in this situation, the biologist's goodwill may be less relevant.

A more atypical example is provided by NASA (National Aeronautics and Space Administration) climatologist Jim Hansen and economist and former IPCC (Intergovernmental Panel on Climate Change)‐member Mark Jaccard (Frid & Quarmby 2012), who chose to be arrested in acts of civil disobedience to publicly condemn governmental inaction regarding climate change (Hansen 2012; Jaccard 2013; Frid 2015). A simplistic view of credibility would discredit these scientists, yet a more complex and richer perspective of credibility acknowledges that these senior scientists demonstrated expertise in their recognition of a mismatch between policy and the urgent need to reduce emissions, goodwill toward future generations and current climate refugees, and trustworthiness (i.e., acting honestly without a hidden agenda). Rather than harming scientific credibility, their arrests delivered the message that Hansen and Jaccard's science‐based concern for human society and biodiversity supersedes potential risks to their careers; thereby, they strengthened public recognition that scientists also are citizens and may reasonably advocate for political action without losing credibility.

Conservation science is about more than material reality; its very existence depends on symbolic realities that emerge from socially constructed values (Soulé 1985; Naess 1986; Moon & Blackman 2014). Peterson et al. (2013:100–101) argue that “To do proper justice to these values in the public sphere requires rhetoric and public processes that are honest about human politics and human relationships with biodiversity.”

Conscious awareness of terminologies or linguistic patterns (Burke 1966; Kao 2013) may enable conservation scientists to more effectively negotiate the scientist–advocate dilemma. With a more nuanced understanding of credibility, conservation scientists are better equipped to recognize existing terminologies and to reframe them in ways that better meet stakeholder expectations. Reconceptualizing credibility as a sociopolitical process that produces only fleeting moments of stability, and then recognizing the multidimensionality of credibility, will not do away with the perceived trade‐offs between science and advocacy, but it will help conservation professionals negotiate them more effectively.

## References

[cobi12558-bib-0001] Alagona PS . 2008 Credibility. Conservation Biology 22:1365–1367. 19076862 10.1111/j.1523-1739.2008.01109.x

[cobi12558-bib-0002] Allen TFH , Tainter JA , Pires JC , Hoekstra TW . 2001 Dragnet ecology—“Just the facts, Ma'am”: The privilege of science in a postmodern world. BioScience 51:475–485.

[cobi12558-bib-0003] Aristotle. 4th century BCE/ 1991 On rhetoric: A theory of civic discourse. Oxford University Press, New York.

[cobi12558-bib-0004] Barry D , Oelschlaeger M . 1996 A science for survival: Values and conservation biology. Conservation Biology 10:905–911.

[cobi12558-bib-0005] Blockstein DE . 2002 How to lose your political virginity while keeping your scientific credibility. BioScience 52:91–96.

[cobi12558-bib-0006] Brosnan DM . 1995 Bridging gaps among ecology, law, and policy. Wildlife Society Bulletin 23:333–337.

[cobi12558-bib-0007] Brussard PF , Tull JC . 2007 Conservation biology and four types of advocacy. Conservation Biology 21:21–24. 17298506 10.1111/j.1523-1739.2006.00640.x

[cobi12558-bib-0008] Burke K . 1966 Language as symbolic action: essays on life, literature, and method. University of California Press, Berkeley.

[cobi12558-bib-0009] Chan KMA . 2008 Value and advocacy in conservation biology: Crisis discipline or discipline in crisis? Conservation Biology 22:1–3. 18254846 10.1111/j.1523-1739.2007.00869.x

[cobi12558-bib-0010] Cohen J . 1968 Weighted kappa: Nominal scale agreement with provision for scaled disagreement or partial credit. Psychological Bulletin 70:213–220. 19673146 10.1037/h0026256

[cobi12558-bib-0011] Corbin J , Strauss A . 2008 Basics of qualitative research: Techniques and procedures for developing grounded theory. Sage, Thousand Oaks, CA.

[cobi12558-bib-0012] Costanza R . 2001 Visions, values, valuation, and the need for an ecological economics. BioScience 51:459–468.

[cobi12558-bib-0013] Cronkhite G , Liska J . 1976 A critique of factor analytic approaches to the study of credibility. Communication Monographs 43:91–107.

[cobi12558-bib-0014] Decker DJ , Roland ES , Nielsen LA , Parsons GR . 1991 Ethical and scientific judgements in management: Beware of blurred distinctions. Wildlife Society Bulletin 19:523–527.

[cobi12558-bib-0015] Freyfogle ET , Newton JL . 2002 Putting science in its place. Conservation Biology 16:863–873.

[cobi12558-bib-0016] Frid A . 2015 Conservation value of paddy wagon currency: civil disobedience by scientists. ConservationBytes: 5 March 2015. Available from http://conservationbytes.com/2012/05/12/conservation‐value‐of‐paddy‐wagon‐currency/#more‐7147.

[cobi12558-bib-0017] Frid A , Quarmby L . 2012 Take direct action on climate inaction. Nature 487:38. 22763536 10.1038/487038a

[cobi12558-bib-0018] Gill RB . 2001 Professionalism, advocacy, and credibility: A futile cycle? Human Dimensions of Wildlife 6:21–32.

[cobi12558-bib-0019] Goodwin J . 2012 What is "responsible advocacy" in science? Good advice Pages 151–161 in Goodwin J, editor. Between scientists and citizens: proceedings of a conference at Iowa State University. Great Plains Society for the Study of Argumentation, Ames, IA.

[cobi12558-bib-0020] Hansen J . 2012 Why I must speak out about climate change. TED: March. Available from https://www.ted.com/people/speakers (accessed March 2015).

[cobi12558-bib-0021] Jaccard M . 2013 The accidental activist: How an energy economist and former government adviser found himself blocking a coal train. The Walrus Available from http://thewalrus.ca/the‐accidental‐activist/ (accessed June 2015).

[cobi12558-bib-0022] Jasanoff S , Markle GE , Petersen JC , Pinch T . 1995 Handbook of science and technology studies. Sage, Thousand Oaks, CA.

[cobi12558-bib-0023] Kao DT. 2013 The impacts of goal orientation, terminology effect, and source credibility on communication effectiveness. Journal of Applied Social Psychology 43:2007–2016.

[cobi12558-bib-0024] Kennedy GA . 1999 Classical rhetoric and its Christian and secular tradition form ancient to modern times. University of North Carolina Press, Chapel Hill, NC.

[cobi12558-bib-0025] Kinchy AJ , Kleinman DL . 2003 Organizing credibility: discursive and organizational orthodoxy on the borders of ecology and politics. Social Studies of Science 33:869–896.

[cobi12558-bib-0026] Krippendorff K . 2013 Content analysis: an introduction to its methodology. Sage Publications, Thousand Oaks, CA.

[cobi12558-bib-0027] Lackey RT . 2007 Science, scientists, and policy advocacy. Conservation Biology 21:12–17. 17298504 10.1111/j.1523-1739.2006.00639.x

[cobi12558-bib-0028] Leff MC . 1980 Interpretation and the art of the rhetorical critic. Journal of Applied Communication Research 44:337–349.

[cobi12558-bib-0029] Macnab J . 1985 Carrying capacity and related slippery shibboleths. Wildlife Society Bulletin 13:403–410.

[cobi12558-bib-0030] Malshe A . 2010 How is marketers' credibility construed within the sales‐marketing interface? Journal of Business Research 63:13–19.

[cobi12558-bib-0031] McCroskey JC . 1997 An introduction to rhetorical communication. Allyn and Bacon, Boston.

[cobi12558-bib-0032] Meffe GK . 2007 Conservation focus: Policy advocacy and conservation science. Conservation Biology 21:11.

[cobi12558-bib-0033] Meffe GK , Viederman S . 1995 Combining science and policy in conservation biology. Wildlife Society Bulletin 23:327–332.

[cobi12558-bib-0034] Meyer JL , Frumhoff PC , Hamburg SP , de la Rosa C . 2010 Above the din but in the fray: environmental scientists as effective advocates. Frontiers in Ecology and the Environment 8:299–305.

[cobi12558-bib-0035] Moon K , Blackman D . 2014 A guide to understanding social science research for natural scientists. Conservation Biology 28:1167–1177. 24962114 10.1111/cobi.12326

[cobi12558-bib-0036] Mooney HA . 2003 The ecology‐policy interface. Frontiers in Ecology and the Environment 1:49.

[cobi12558-bib-0037] Morrison ML , Block WM , Strickland MD , Collier BA , Peterson MJ . 2008 Wildlife study design. Springer, New York.

[cobi12558-bib-0038] Murphy DD , Noon BR . 2007 The role of scientists in conservation planning on private lands. Conservation Biology 21:25–28. 17298507 10.1111/j.1523-1739.2006.00642.x

[cobi12558-bib-0039] Naess A . 1986 Intrinsic value: Will the defenders of nature please rise? Pages 504‐515 in Soulé ME, editor. Conservation biology: the science of scarcity and diversity. Sinauer Associates, Sunderland, MA.

[cobi12558-bib-0040] Nelson MP , Vucetich JA . 2009 On advocacy by environmental scientists: what, whether, why, and how. Conservation Biology 23:1090–1101. 19459889 10.1111/j.1523-1739.2009.01250.x

[cobi12558-bib-0041] Newell SJ , Goldsmith RE . 2001 The development of a scale to measure perceived corporate credibility. Journal of Business Research 52:235–247.

[cobi12558-bib-0042] Noss RF . 2007 Values are a good thing in conservation biology. Conservation Biology 21:18–20. 17298505 10.1111/j.1523-1739.2006.00637.x

[cobi12558-bib-0043] Peterson MJ . 2009 An ecologist's response to the SIU campaign: the role of natural scientists in environmental movements Pages 361–387 in Endres D, Sprain L, Peterson TR, editors. Social movement to address climate change: local steps for global action. Cambria Press, Amherst, NY.

[cobi12558-bib-0044] Peterson MJ , Silvy NJ . 1994 Spring precipitation and fluctuations in Attwater's prairie‐chicken numbers: hypotheses revisited. Journal of Wildlife Management 58:222–229.

[cobi12558-bib-0045] Peterson MJ , Silvy NJ . 1996 Reproductive stages limiting productivity of the endangered Attwater's prairie chicken. Conservation Biology 10:1264–1276.

[cobi12558-bib-0046] Peterson MN , Peterson MJ , Peterson TR , Leong K . 2013 Why transforming biodiversity conservation conflict is essential and how to begin. Pacific Conservation Biology 19:94–103.

[cobi12558-bib-0047] Peterson MN , Peterson TR , Birckhead JL , Leong K , Peterson MJ . 2010 Rearticulating the myth of human‐wildlife conflict. Conservation Letters 3:74–82.

[cobi12558-bib-0048] Peterson TR , Horton CC . 1995 Rooted in the soil: how understanding the perspectives of landowners can enhance the management of environmental disputes. Quarterly Journal of Speech 81:139–166.

[cobi12558-bib-0049] Peterson TR , Witte K , Enkerlin‐Hoeflich E , Espericueta L , Flora JT , Florey N , Loughran T , Stuart R . 1994 Using informant directed interviews to discover risk orientation: how formative evaluations based in interpretive analysis can improve persuasive safety campaigns. Journal of Applied Communication Research 22:199–215.

[cobi12558-bib-0050] Platt JR . 1964 Strong inference: certain systematic methods of scientific thinking may produce much more rapid progress than others. Science 146:347–353. 17739513 10.1126/science.146.3642.347

[cobi12558-bib-0051] Popper KR . 1959 The logic of scientific discovery. Hutchinson, London.

[cobi12558-bib-0052] Popper KR . 1962 Conjectures and refutations: the growth of scientific knowledge. Basic Books, New York.

[cobi12558-bib-0053] Rhee EY , Fiss PC . 2014 Framing controversial actions: regulatory focus, source credibility, and stock market reaction to poison pill adoption. Academy of Management Journal 57:1734–1758.

[cobi12558-bib-0054] Rudd MA . 2011 How research‐prioritization exercises affect conservation policy. Conservation Biology 25:860–866. 21790784 10.1111/j.1523-1739.2011.01712.x

[cobi12558-bib-0055] Ruggiero LF . 2010 Scientific independence and credibility in sociopolitical processes. Journal of Wildlife Management 74:1179–1182.

[cobi12558-bib-0056] Rutberg AT . 2001 Why state agencies should not advocate hunting or trapping. Human Dimensions of Wildlife 6:33–37.

[cobi12558-bib-0057] Sah S , Moore DA , MacCoun RJ . 2013 Cheap talk and credibility: The consequences of confidence and accuracy on advisor credibility and persuasiveness. Organizational Behavior and Human Decision Processes 121:246–255.

[cobi12558-bib-0058] Sallenave R , Cowley DE . 2006 Science and effective policy for managing aquatic resources. Reviews in Fisheries Science 14:203–210.

[cobi12558-bib-0059] Salzman JE . 1989 Scientists as advocates: The Point Reyes Bird Observatory and gill netting in central California. Conservation Biology 3:170–180.

[cobi12558-bib-0060] SAS Institute . 2012. SAS 9.3. SAS Institute, Cary, North Carolina.

[cobi12558-bib-0061] Scott JM , et al. 2007 Policy advocacy in science: Prevalence, perspectives, and implications for conservation biologists. Conservation Biology 21:29–35. 17298508 10.1111/j.1523-1739.2006.00641.x

[cobi12558-bib-0062] Silvy NJ , Peterson MJ , Lopez RR . 2004 The cause of the decline of pinnated grouse: The Texas example. Wildlife Society Bulletin 32:16–21.

[cobi12558-bib-0063] Soulé ME . 1985 What is conservation biology? BioScience 35:727–734.

[cobi12558-bib-0064] Soulé ME . 1986 Conservation biology and the "real world" Pages 1‐12 in Soulé ME, editor. Conservation biology: the science of scarcity and diversity. Sinauer Associates, Sunderland, MA.

[cobi12558-bib-0065] Thompson B . 1995 Overcoming gaps in implementing policy: Influencing policy is just a means to an end, not the end! Wildlife Society Bulletin 23:317–318.

[cobi12558-bib-0066] Tracy CR , Brussard PF . 1996 The importance of science in conservation biology. Conservation Biology 10:918–919.

[cobi12558-bib-0067] Vohland K , Mlambo MC , Horta LD , Jonsson B , Paulsch A , Martinez SI . 2011 How to ensure a credible and efficient IPBES? Environmental Science & Policy 14:1188–1194.

[cobi12558-bib-0068] Wilhere GF . 2012 Inadvertent advocacy. Conservation Biology 26:39–46. 22280324 10.1111/j.1523-1739.2011.01805.x

[cobi12558-bib-0069] Yamamoto YT . 2012 Values, objectivity and credibility of scientists in a contentious natural resource debate. Public Understanding of Science 21:101–125. 22530490 10.1177/0963662510371435

